# Effects of contact ultrasound coupled with infrared radiation on drying kinetics, water migration and physical properties of beef during hot air drying

**DOI:** 10.1016/j.ultsonch.2024.106978

**Published:** 2024-07-03

**Authors:** Jiahua Gao, Siyu Cheng, Xianming Zeng, Xiaomei Sun, Yun Bai, Songmei Hu, Jianping Yue, Xiaobo Yu, Minwei Zhang, Xinglian Xu, Minyi Han

**Affiliations:** a Key Laboratory of Meat Processing and Quality Control, Ministry of Education, Nanjing Agricultural University, Nanjing 210095, China; b Wens Foodstuff Group Co., Ltd., Yunfu 527400, China; c Emin County Xinda Tongchuang Bioengineering Co., Ltd., Tacheng 834600, China; d Guangdong Testing Institute of Product Quality Supervision, Shunde 528300, China

**Keywords:** Contact ultrasound, Infrared radiation, Drying kinetics, Water migration, Protein denaturation, Physical properties

## Abstract

Drying, as a critical step in the production of air-dried beef, has a direct impact on the quality of the final product. Innovatively, a composite system incorporating contact ultrasound (CU) and infrared radiation (IR) as auxiliary measures within a hot air drying (HAD) framework was built in this research, and the effects of these techniques on the drying kinetics, protein denaturation, and moisture transformation of air-dried beef were investigated. In comparison to HAD treatment, the integrated CU and IR (CU-IRD) system displayed marked enhancements in heat and moisture transport efficiency, thereby saving 36.84% of time expenditure and contributing favorably to the improved moisture distribution of the end-product. This was mainly ascribed to the denaturation of myosin induced by IR thermal effect and the micro-channel produced by CU sponge effect, thus increasing T_2_ relaxation time and the proportion of free water. In conclusion, the composite system solved the problem of surface hardening and reduces hardness and chewiness of air-dried beef by 40.42% and 45.25% respectively, but inevitably increased the energy burden by 41.60%.

## Highlights

• CU-IRD displayed marked enhancements in heat and moisture transport efficiency.
• CU and IR increased myosin denaturation and water fluidity.
• CU destroyed the fiber integrity and improved the texture.
• CU-IRD showed the shortest drying time and higher drying uniformity.

## 1 Introduction

Air-dried beef, a typical natural fermented cured meat product in China, undergoes dynamic shifts in its microbiological ecosystem throughout its production process [1]. In the extended fermentation period of air-drying, the meat accumulated a diverse array of microbial populations through natural selection and prolonged domestication, including beneficial microorganisms such as *lactobacillus* and *yeast* species [2]. However, the inherent openness of the traditional air-drying method renders the meat products vulnerable to infestation by insects and environmental contaminants, leading to significant challenges to quality control and standardized production.

In industrial practice, convection drying or hot air drying techniques operating around 35 °C are widely adopted as the mainstream processing methods for air-dried meat products [3]. Given the low thermal conductivity of foodstuffs, limited heat transfer within the product results in poor energy utilization efficiency and extended drying times during convection heating [4]. It is noteworthy that prolonged exposure to high temperatures not only accelerates non-enzymatic browning reactions and other heat-induced transformations, but also leads to degradation in multiple aspects of food quality, including fat oxidation, discoloration, reduction in vitamin content, and destruction of amino acids [5]. In light of these considerations, exploring and implementing more advanced drying technologies is of critical importance to enhance drying efficiency while mitigating detrimental effects associated with heat treatment.

In recent years, infrared (IR) technology has increasingly emerged as a promising alternative to traditional drying methods due to its versatility, rapid heating and drying response, ease of installation, and relatively low initial investment costs [6]. IR is an electromagnetic wave that originates from a heat source and lies between the visible light and microwave spectra, with wavelengths ranging from 0.76 to 1000 μm [7]. When applied to materials with high water content, the unique electromagnetic penetration properties allow the energy to be absorbed by molecules at different depths within the material, raising molecular vibrational energy levels and creating a relatively uniform temperature distribution inside the material [8]. Notably, IR emission and propagation are independent of the medium, thereby offering higher heat transfer efficiency and lower energy losses compared to conductive and convective heat transfer methods.

In the drying process of food products, contact ultrasound (CU) has attracted attention as an effective strategy to accelerate mass transfer, which operating principle primarily involves non-thermal mechanisms that intervene in internal and surface mass transfer resistances within solid materials [9]. On one hand, the high-frequency vibrations imposed by ultrasound induce a series of rapid and periodic compression and expansion (sponge effect) within the solid medium, which is driven by the mechanical action of ultrasound waves [10]. This alternating stress improves intraparticle diffusion of water molecules and creates micro-channels within the material, thereby enhancing the ease with which moisture migrates out. On the other hand, high-intensity acoustic waves have the potential to induce cavitation of water molecules inside food materials, which is beneficial for removing tightly bound water [11]. Concurrently, the cavitation phenomenon and accompanying physical and chemical effects include microcurrents, turbulence, localized extreme high pressures and high temperatures [12]. This process may deform intercellular and extracellular structures, thereby reducing the diffusion boundary layer and increasing the rate of water transport to the external environment [13]. To our knowledge, the application of CU and IR in low-temperature beef drying remains unexplored.

In the present study, CU with IR were combined to develop a novel composite drying system, aiming to optimize the processing outcomes of hot air drying on beef products. The mass transfer mechanism during the drying process was quantitatively described by mathematical models. Combined with water migration, protein denaturation and physical properties, the strengthening effect of this process on drying was investigated.

## 2 Materials and methods

### 2.1 Sample preparation

In this study, the *longissimus dorsi* (LD) were purchased from a local commercial abattoir (Yurun Meat Processing Company, Nanjing, China) within 24 h post-mortem, which were obtained from six 3-year-old cattle. Visible fat and fascia were removed from the surface, and the muscle was then divided into four portions and stored at −18 °C in a refrigerated facility to accommodate different treatments. Prior to experimentation, the meat was thawed at a temperature of 4 °C for 24 h and subsequently cut into cubes measuring 15 mm × 15 mm × 40 mm with a weight of approximately 10 ± 0.6 g, ensuring that the longest side was aligned with the direction of muscle fibers. To minimize potential interference caused by storage duration on experimental parameters, the duration of a single experimental run lasted no longer than one week.

### 2.2 Experimental equipment and drying procedures

**Fig.1:**
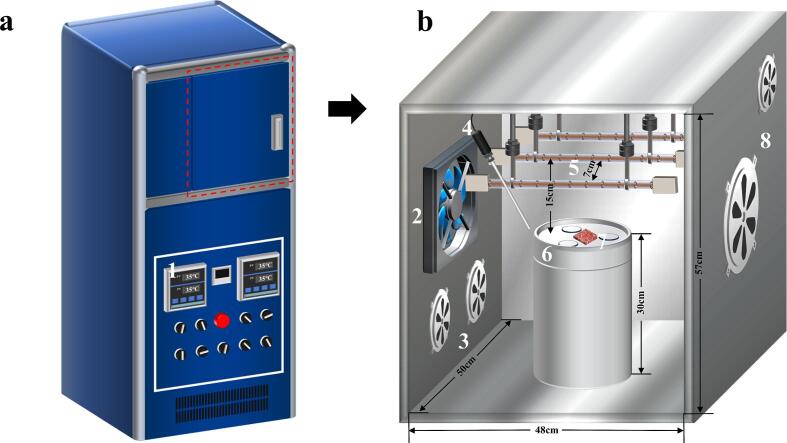
Schematic diagram of drying equipment (a. oven shell; b. internal structure) 1.control panel, 2. axial flow fan, 3. air inlet, 4. K-type thermocouple, 5. infrared lamps, 6. ultrasonic vibration plate, 7. beef sample, 8. air outlet.

The drying experiments were carried out in a self-assembled combined dryer. A schematic diagram of the experimental setup is presented in Fig. 1, in which a 25 cm diameter CU vibrating plate operating at a frequency of 20 kHz (BOS2015ZKB, Shangjia Biotechnology Co., Ltd., China) was installed. The ultrasonic mode was programmed to operate in an intermittent pattern (5 s on, 5 s off).The actual ultrasonic power distribution on the surface was determined to be 12.6 W/dm^2^ according to the calorimetric method proposed by Mason et al. [14].

In order to provide uniform radiation density, the infrared heating apparatus consists of three identical infrared lamps (IR74-115–300, Guangzhiyang Photoelectricity Technology Co., Ltd., China) connected in series, whose actual voltage and power are regulated by an external voltage regulator (STG-500VA, Chengqiang Electricity Co., Ltd., China). Each lamp's specifications were as follows: rated voltage 74 V, rated power 115 W, total length 300 mm and heated length 230 mm. The final temperature of the sample was around 35 °C without large fluctuations when a voltage of 120 V was applied (the actual power is 140 W) as measured in the preliminary experiments.

The air conditioning unit comprised an axial flow fan equipped with a preheating mechanism (FP-108EX-S1-B, Taiwan Sanxie Industrial Co., Ltd., China) along with a K-type thermocouple (TB604-1, Suzhou Tasi Electronics Co., Ltd., China). The K-type thermocouple was connected to the control panel of the dryer, enabling continuous monitoring and maintenance of a constant drying air temperature within the system (set at 35 °C in this study).

Table 1 demonstrates the different combinations of drying conditions used in this experiment, with or without the use of CU and/or IR. The initial moisture content of the beef samples was measured as 74.82 ± 0.63 % on a wet basis (w. b.) using the method as outlined by Sánchez-Torres et al. [15], which corresponds to approximately 297.14 % on a dry basis (d. b.). The experiment was extended to 50 % weight loss, which is expected to reduce the moisture content of the samples to 50 % (w. b.), with a weighing interval of 30 min. During the entire duration of the drying, the beef samples were placed on the ultrasonic vibration plate for treatment. All experiments were performed in triplicate.

**Table 1:** Drying conditions and the corresponding codes used in this research.

Sample code	Contacting ultrasound (CU)	Infrared (IR)	Hot air
HAD	−	−	+
CU-HAD	+	−	+
IRD	−	+	+
CU-IRD	+	+	+

### 2.3 Internal temperature change

The temperature variations within the beef during the drying process were continuously monitored and recorded by a temperature data logger (RDXL 12SD, Omega Therapeutics Inc., USA). For this purpose, randomly selected samples were subjected to internal temperature measurements with the needle probe of a K-type thermocouple inserted at the core of each sample. The temperature readings were taken at an interval of 5 s for each measurement instance.

### 2.4 Moisture content, moisture ratio and drying rate

The dry basis moisture content, moisture ratio and drying rate at each time point were calculated and analyzed separately.

(1) $${\text{M}}_{\text{t}}=\frac{{\text{(m}}_{\text{t}}\text{-}{\text{m}}_{\text{0}}\text{X)}}{{\text{m}}_{\text{0}}\text{X}}\times \text{100\%}$$

where m_0_, m_t_ are the initial and drying to t mass of beef, respectively, g; X, M_t_ are the moisture content (% d. b.) of beef at the initial and dried to the moment of t, respectively.

The moisture ratio (MR) of beef was obtained from equation (2).

(2) $$\text{MR =}\frac{{\text{M}}_{\text{t}}\text{-}{\text{M}}_{\text{e}}}{{\text{M}}_{\text{0}}\text{-}{\text{M}}_{\text{e}}}$$

where the subscripts t, e and 0 describe the instantaneous, equilibrium and initial values, respectively. In the present study, the equation has been simplified to MR = M_t_ /M_0_ due to the fact that M_e_ is significantly smaller in comparison to either M_t_ or M_0_. The drying rate (DR) of beef is the amount of moisture lost per unit of time and is calculated by the formula (3).

(3) $$\text{DR=}\frac{{\text{M}}_{\text{t}}\text{-}{\text{M}}_{\text{t+}\Delta \text{t}}}{\Delta \text{t}}$$

where M_t_ and M_t + Δt_ are the moisture content (% d. b.) of the sample at t and t + Δt, respectively; t and Δt are both drying times, min.

### 2.5 Effective water diffusion coefficient and kinetic modeling

For materials undergoing a falling-rate drying stage, the underlying mechanism can be described by Fick's second law of diffusion [16]. Under the assumptions that the beef is homogeneous and isotropic, and that the resistance to moisture flow within the material is uniformly distributed in the context of thin-layer drying theory, the governing equation is represented by Equation (4).

(4) $$\text{MR =}\frac{\text{8}}{\pi^{\text{2}}}\sum_{\text{n=0}}^{\infty }\frac{\text{1}}{{\text{(2n+1)}}^{\text{2}}}\times \text{exp}\left[\frac{\text{-}{\text{(2n+1)}}^{\text{2}}\pi^{\text{2}}{\text{D}}_{\text{eff}}\text{t}}{\text{4}{\text{L}}^{\text{2}}}\right]$$

Where D_eff_ is the effective moisture diffusion coefficient (m^2^/min); L is the thickness of half specimen (m); t is the drying time (min); n = 0, 1, 2….…...

This equation involved has a complex structure and takes the form of an infinite series. When considering the long-term conditions of the drying process, the case of considering only the zero-order term (n = 0) was chosen [17], i.e., the moisture diffusion coefficient can be approximated by Eq. (5):

(5) $$\text{MR =}\frac{\text{8}}{\pi^{\text{2}}}\;\text{exp}\left[\frac{\text{-}\pi^{\text{2}}{\text{D}}_{\text{eff}}\text{t}}{\text{4}{\text{L}}^{\text{2}}}\right]$$

By performing a logarithmic transformation operation on both sides of the original nonlinear equation, a new linear expression, Equation (6), can be derived. By analyzing and calculating the slope of the straight line represented by Equation (6), the D_eff_ of water can be effectively extracted.

(6) $$\text{ln}\left(\text{MR}\right)=\text{ln}\text{(}\frac{\text{8}}{\pi^{\text{2}}}\text{)}\text{-}\frac{\pi^{\text{2}}{\text{D}}_{\text{eff}}}{\text{4}{\text{L}}^{\text{2}}}\text{Â·t}$$

Within this research, a comprehensive dataset has been collected that encompasses the relationship between time and MR during the drying process. As depicted in Table 2, these empirical data have been subjected to simulation fitting using Fick's second law as the fundamental theoretical model, alongside ten additional empirical models, with the aim to holistically and multidimensionally unravel and expound upon the kinetic laws governing the drying process.

**Table 2:** Theoretical and empirical models and related parameters used in this study.

**Models**	**Drying** **method**	**Constants and coefficients**	**Statistical parameters**		
			**RMSE**	**SSE**	**R^2^**
**Fick's second law** $\text{MR =}\frac{\text{8}}{\pi^{\text{2}}}\;\text{exp}\left[\frac{-\pi^{\text{2}}{\text{D}}_{\text{eff}}\text{t}}{\text{4}{\text{L}}^{\text{2}}}\right]$	HAD	D_eff_ = 3.880 × 10^-8^	0.0516	0.0479	0.9717
	CU-HAD	D_eff_ = 4.675 × 10^-8^	0.0571	0.0489	0.9697
	IRD	D_eff_ = 5.095 × 10^-8^	0.0471	0.0288	0.9762
	CU-IRD	D_eff_ = 5.944 × 10^-8^	0.0464	0.0237	0.9759
**Newton（Lewis）** MR = exp(−kt)	HAD	k = 0.0023	0.0378	0.0257	0.9545
	CU-HAD	k = 0.0028	0.0353	0.0187	0.9622
	IRD	k = 0.0031	0.0418	0.0228	0.9382
	CU-IRD	k = 0.0037	0.0498	0.0273	0.9064
**Page** MR = exp(−k·t^n^)	HAD	k = 0.0133n = 0.7053	0.0109	0.0020	0.9960
	CU-HAD	k = 0.0089n = 0.7868	0.0097	0.0013	0.9971
	IRD	k = 0.0123n = 0.7508	0.0079	0.0007	0.9980
	CU-IRD	k = 0.0181n = 0.7037	0.0115	0.0013	0.9954
**Modified Page** MR = exp(−(k·t)^n^)	HAD	k = 0.0022n = 0.7053	0.0109	0.0020	0.9960
	CU-HAD	k = 0.0025n = 0.7868	0.0097	0.0013	0.9971
	IRD	k = 0.0028n = 0.7246	0.0076	0.0007	0.9980
	CU-IRD	k = 0.0033n = 0.7037	0.0115	0.0013	0.9954
**Henderson and Pabis** MR = a exp(−k·t)	HAD	a = 0.9169 k = 0.0020	0.0245	0.0102	0.9819
	CU-HAD	a = 0.9184 k = 0.0023	0.0218	0.0067	0.9854
	IRD	a = 0.8963 k = 0.0026	0.0216	0.0056	0.9848
	CU-IRD	a = 0.8762 k = 0.0030	0.0270	0.0073	0.9751
**Logarithmic** MR = a exp(−kt) + b	HAD	a = 0.7373 k = 0.0040 b = 0.2534	0.0033	0.0002	0.9997
	CU-HAD	a = 0.7583 k = 0.0049 b = 0.2560	0.0062	0.0005	0.9990
	IRD	a = 0.6929 k = 0.0051 b = 0.2608	0.0061	0.0004	0.9988
	CU-IRD	a = 0.6909 k = 0.0071 b = 0.2820	0.0068	0.0004	0.9986
**Two-term** MR = a exp(−k_1_t) + b exp(−k_2_t)	HAD	a = 0.5080 k_1_ = 0.0056 b = 0.4949 k_2_ = 0.0009	0.0019	5.651 × 10^-5^	0.9999
	CU-HAD	a = 0.7564 k_1_ = 0.0018 b = 0.2742 k_2_ = 0.0125	0.0010	1.183 × 10^-5^	1.000
	IRD	a = 0.7195 k_1_ = 0.0019 b = 0.2861 k_2_ = 0.0130	0.0022	4.664 × 10^-5^	0.9999
	CU-IRD	a = 0.3411 k_1_ = 0.0169 b = 0.6820 k_2_ = 0.0020	0.0015	1.872 × 10^-5^	0.9999
**Two-term exponential** MR = a exp(−kt) +(1 − a) exp(−kat)	HAD	a = 0.1767 k = 0.0102	0.0137	0.0032	0.9943
	CU-HAD	a = 0.1670 k = 0.0125	0.0108	0.0016	0.9965
	IRD	a = 0.1587 k = 0.0154	0.0158	0.0030	0.9918
	CU-IRD	a = 0.1626 k = 0.0178	0.0242	0.0058	0.9800
**Wang and Singh** MR = 1 + a·t + b·t^2^	HAD	a = -0.0025b = 2.398 × 10^-6^	0.0196	0.0066	0.9884
	CU-HAD	a = -0.0029b = 3.280 × 10^-6^	0.0184	0.0048	0.9904
	IRD	a = -0.0033b = 4.335 × 10^-6^	0.0267	0.0085	0.9769
	CU-IRD	a = -0.0042b = 6.674 × 10^-6^	0.0305	0.0093	0.9683
**Approximation of diffusion** MR = a exp(−kt) + (1 − a) exp(−kbt)	HAD	a = 0.5277 k = 0.0053 b = 0.1499	0.0019	5.959 × 10^-5^	0.9999
	CU-HAD	a = 0.3166 k = 0.0088 b = 0.1794	0.0036	0.0002	0.9996
	IRD	a = 0.2927 k = 0.0122 b = 0.1533	0.0021	5.004 × 10^-5^	0.9999
	CU-IRD	a = 0.3518 k = 0.0142 b = 0.1335	0.0025	5.687 × 10^-5^	0.9998
**Weibull** $\text{MR = exp}\left[-{\left(\frac{\text{t}}{\alpha }\right)}^{\beta }\right]$	HAD	α = 457.0 β = 0.7053	0.0109	0.0020	0.9960
	CU-HAD	α = 375.4 β = 0.7720	0.0124	0.0022	0.9954
	IRD	α = 361.9 β = 0.7246	0.0076	0.0007	0.9980
	CU-IRD	α = 299.3 β = 0.7037	0.0115	0.0013	0.9954

Matlab R2022a (MathWorks Inc., USA) was utilized for the parameter estimation and fitting analysis of the drying model. Based on the optimal discriminators of model performance, the model with the highest coefficient of determination (R^2^), the lowest root mean square error (RMSE), and the smallest sum of squared errors (SSE) was selected as the most suitable model for describing the drying process. The precise values of the above evaluation indexes were rigorously deduced and calculated by the respective corresponding mathematical formulas:

（7） $${\text{R}}^{\text{2}}\;\text{= 1 -}\;\left[\frac{\sum_{\text{i = 1}}^{\text{N}}{\left({\text{y}}_{\text{exp, i}}\text{-}{\;\text{y}}_{\text{pred, i}}\right)}^{\text{2}}}{\sum_{\text{i = 1}}^{\text{N}}{\left({\text{y}}_{\text{exp, i}}\text{-}\;{\text{y}}_{\text{mean}}\right)}^{\text{2}}}\right]$$

（8） $$\text{SSE =}\;\sum_{\text{i=1}}^{\text{N}}{\left({\text{y}}_{\text{exp, i}}\text{-}{\;\text{y}}_{\text{pred, i}}\right)}^{\text{2}}$$

（9） $$\text{RMSE =}{\left(\frac{\text{SSE}}{\upsilon }\right)}^{\frac{\text{1}}{\text{2}}}\text{, where}\;\upsilon \;\text{= N - m}$$

where N is the number of experimental data points, m is the number of fitted coefficients in each equation. And y_mean_ is the mean of the experimental values, while y_exp, i_ and y_pred, i_ are the i^th^ experimental and predicted values, respectively.

### 2.6 Energy consumption

In the present study, energy consumption is the aggregate amount of energy consumed by all electrical components within the drying systems, encompassing the fan, preheating apparatus, infrared lamps, and ultrasound equipment. An energy meter apparatus (DL333502, Deli Group Co., Ltd., China) was utilized to directly document energy expenditure in kWh. This device is equipped with all necessary measurement functions to monitor current, voltage, and power in the electrical devices.

### 2.7 Low field-nuclear magnetic resonance (LF-NMR)

In order to facilitate the comparison of the content and distribution of water molecules in the original, early-stage, and late-stage dried samples and predict their changing trends, a LF-NMR analysis system (NMI20-040H-I, Niumag Electric Corporation, China) was used to perform the transversal relaxation of samples undergoing different stages of water loss (0 %, 20 %, and 40 % weight-loss, respectively), using the method of Li et al. [18]. The Carr-Purcell-Meiboom-Gill (CPMG) sequence parameters were set as follows: spectrometer frequency (SF) of 40 MHz, spectral width (SW) 200 kHz, offset frequency (O1) 648.41 kHz, waiting time (TW) 3000 ms, radio frequency delay time (RFD) 0.002 ms, 90° pulse width (P_1_) 7.00 μs, 180° pulse width (P_2_) 13.52 μs, echo time (TE) 0.300 ms, number of echoes (NECH) 5000, and four data accumulation acquisitions.

### 2.8 Differential scanning calorimetry

The denaturation behavior of proteins was investigated using a differential scanning calorimeter (DSC 800, Perkin Elmer Inc., USA). The samples were weighed 10 ∼ 12 mg, sealed in aluminum trays, and put into the instrument for the determination with the following parameters: the heating temperature range was 20 ∼ 100 °C, and the temperature increase rate was 10 °C·min^−1^.

### 2.9 Surface hydrophobicity

The extraction of crude protein was adapted from the method described by Zhang et al. [19]. Minced meat sample of 3 g was mixed with 50 mmol/L K_2_HPO_4_/KH_2_PO_4_ buffer solution (pH = 7), and homogenized twice at 10,000 rpm for 30 s each time. The homogenate was then centrifuged at 10,000 × g using a high-speed freezing centrifuge (Avanti JXN-26, Beckman Coulter Inc., USA) for 10 min at 4 °C, after which the supernatant was collected and its protein concentration was adjusted to 1 mg/mL.

Following the protocol outlined by Rao et al. [20], the surface hydrophobicity of myofibrillar proteins was characterized using the binding capacity of the hydrophobic dye bromophenol blue (BPB). BPB solution (1 mg/mL) of 40 μL was added to 2 mL of the myofibrillar protein suspension and thoroughly mixed. Similarly, BPB solution was directly introduced into phosphate buffer (without myofibrillar proteins) to serve as a control. Both the control and treated samples were subsequently centrifuged at 4,000 × g for 15 min at 4 °C. The supernatants were collected, and absorbance was measured at 595 nm by microplate reader (Spark, Tecan Group Corporation, Switzerland). The degree of surface hydrophobicity was quantified based on the total bound BPB. The specific calculation formula was as follows:

$$TotalBoundBPB\left(\mu g\right)=40\mu g\times \left(OD_{\mathrm{control}}-OD_{\mathrm{sample}}\right)/OD_{\mathrm{control}}$$

where OD_control_ and OD_sample_ are the absorbance of control and sample group, respectively.

### 2.10 Magnetic resonance imaging (MRI)

Two-dimensional proton density imaging analysis was implemented on samples at the original, mid-drying, and end point of the sample (specifically represented by 0 %, 25 %, and 50 % weight loss) using a MRI analyzer (NMI20-040H-I, Niumag Electric Corporation, China) at a constant temperature of 32 °C. The experiments employed a specific pulse sequence configuration based on the SPIN-ECHO sequence, which included the following parameters: field of view (FOV) set to 60 mm × 60 mm, repetition time (TR) of 500 ms, spin echo time (TE) of 20 ms, and number of repetitions (NS) of four. For each specimen, a central slice with a thickness of 2 mm was selected for analysis.

### 2.11 Texture profile analysis

Following the end of the drying process, the samples were cut along the vertical muscle fiber direction into cubic specimens measuring 10 mm × 10 mm × 10 mm. These samples were subjected to texture profile analysis using a texture analyser (TA-XT Plus, Stable Micro Systems Corporation, England) to determine their hardness, springiness, cohesiveness, and chewiness. The testing conditions were as follows: the P5 probe was employed, with a pre-test speed of 2.0 mm/s, a test speed of 2.0 mm/s, and a post-test rate of 10.0 mm/s. The compression ratio was set at 40 %, the trigger force was 5.0 g, and a two-cycle compression test was then carried out with an interval of 5 s.

### 2.12 Microstructure

Scanning electron microscopy (SEM) was employed to examine the muscle fiber structural characteristics of fresh and dried samples. Initially, the samples were sectioned into cubic blocks measuring 5 mm × 5 mm × 5 mm and fixed by immersion in a 2.5 % glutaraldehyde solution at 4 °C for 24 h. The dehydration was then performed using a series of solutions of increasing ethanol concentration (30 %, 50 %, 70 %, 80 %, 90 % and 100 %). Following this, the specimens were freeze-dried under −40 °C conditions to remove residual moisture. The dried samples then underwent sputter coating with gold before being observed microscopically using a scanning electron microscope (MIRA4, Tescan Co., Ltd., China).

### 2.13 Statistical analysis

Means of the data were compared by one-way analysis of variance (ANOVA) using SAS 8.0 (SAS Institute, USA). For this purpose, significance was defined as P = 0.05 and Duncan's multiple comparisons (Duncan's test) were used. All experiments were repeated three times.

## 3 Results and discussion

### 3.1 Internal temperature change

**Fig.2:**
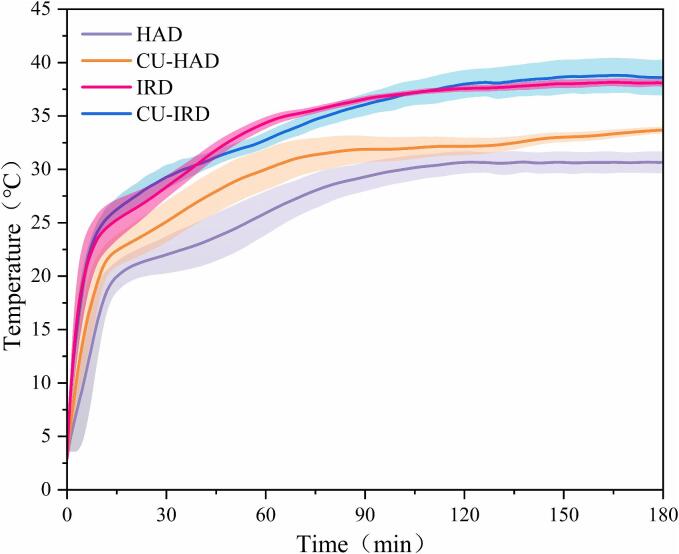
Temperature evolution pattern of beef under different drying conditions.

The temperature evolution patterns of beef under various drying conditions are depicted in Fig. 2. As expected, the sample temperatures in all treatment combinations initially escalated rapidly before stabilizing and oscillating within a range of 30 to 40 °C. Notably, variations were observed in the rate of temperature increase and the eventual steady-state temperature among different processing methods.

During conventional hot air drying, heat transfer occurs as a process that commences from the drying medium onto the sample surface and subsequently penetrates into the interior of the sample, primarily through conduction and diffusion of substances such as water, fat, and other liquid and gaseous materials [21]. Due to the relatively poor thermal conductivity of food matrices, a substantial amount of heat is absorbed at the sample surface, while limited heat is transferred inward. Consequently, in the HAD group, samples exhibited a slower rate of temperature rise, with a characteristic temperature distribution that decreased gradually from the exterior to the interior, consistently remaining below the temperature of the drying medium.

Evidently, the introduction of ultrasound in hot air drying accelerated the heat conduction process, leading to a marked distinction between the temperature curves of the HAD and CU-HAD groups. A feasible explanation is that cavitation bubble implosions generated by ultrasound created intense mechanical agitation at phase interfaces, which helped disturb the working fluid and reduces the thickness of both thermal boundary and fluid boundary layers [22]. As a result, thermal and fraction resistance were diminished, thereby enhancing overall heat transfer performance [23]. Additionally, several studies have also captured the thermal effects of ultrasound [24], [25]. The absorption of ultrasound energy by materials converted it into heat energy, causing a significant rise in temperature. Notably, the temperature curves of the IRD and CU-IRD groups show considerable similarity, suggesting that the mechanical and thermal effects of ultrasound were not prominently evident in the CU-IRD group. This might be due to the substantial contribution of infrared radiation to the sample's thermal energy, causing the proportion of mechanical and thermal energy provided by ultrasound relative to the total medium energy to decrease significantly [9].

The infrared-irradiated samples were significantly different from the other groups in terms of rapid heat transfer rate and higher endpoint temperature, which is related to the mode of action of infrared radiation, i.e., absorption and penetration properties. Regarding absorption properties, specific groups within the samples preferentially absorbed infrared radiation in the 3200–3500 cm^−1^ (2.8–3.1 μm) and 1500–1700 cm^−1^ (5.9–6.7 μm) spectral bands (as depicted in Fig. S1). Specifically, the absorption peak in the 3200–3500 cm^−1^ region reflects the abundant moisture content in beef, resulting from O-H bond stretching vibrations [26].Multiple absorption peaks observed between 2650 and 2950 cm^−1^ predominantly stem from C-H bond stretching in various organic components. As for the two pronounced absorption peaks around 1656 cm^−1^ and 1545 cm^−1^ are mainly associated with the stretching of C = O, N-C = O, and N-H bonds present in peptides [27].Concerning penetration, Onwude et al. [28] reported that infrared radiation can penetrate the interior of the sample, increasing the internal heat flux and raising the temperature. Ordinarily, the penetration depth of infrared radiation in a given material is not a fixed constant but rather dynamically adjusts with changes in wavelength [29]. During this period, heat accumulated internally within the sample and transferred towards the surface, contributing significantly more heat than what would be transferred through conduction from the external hot air.

### 3.2 Drying kinetics

**Fig.3:**
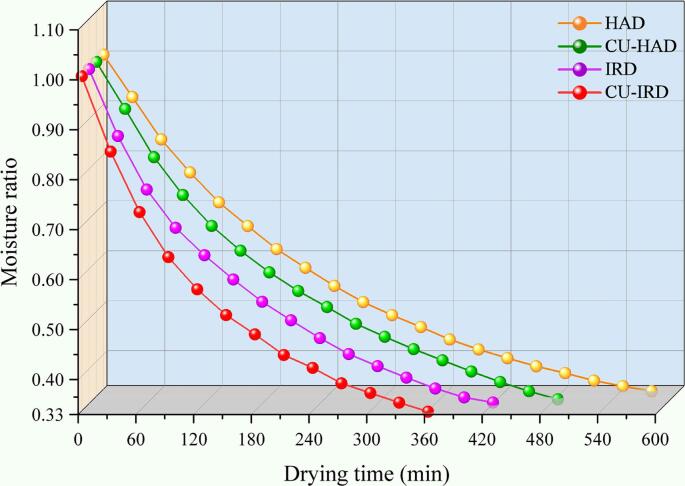
Curve of beef moisture ratio with time under different drying conditions.

In the present study, the moisture ratio (MR) of beef over time under various drying conditions was determined and presented in Fig. 3. It is evident that MR follows an exponential decline pattern, which aligns with the drying characteristics observed in numerous agricultural products [30], [31]. As drying proceeds, the decreasing trend of MR slowed down, which corresponded to the weakening of moisture dissipation. As described by Başlar et al. [32], the decrease in moisture content during drying is accompanied by an increase in protein concentration and the formation of a gel matrix by thermal denaturation, which contribute to increased internal mass transfer resistance.

Addressing this problem, the application of CU effectively accelerated water diffusion, reducing the drying time from 570 min to 480 min. This optimization can be explained by the hypothesis that ultrasound imparted a certain amount of energy into the solid matrix, thereby enhancing water mobility and mitigating internal mass transfer resistance [33]. The energy from the ultrasound caused rapid contraction and expansion of the beef tissue, which reduced adsorption between water molecules and muscle fiber proteins, thus improving water mobility [34]. Concurrently, the high-frequency vibrations induced by ultrasound facilitated the enlargement of mass transfer pathways, lowering internal diffusional resistance, and promoting outward migration of internal water [35].

In comparison to the application of CU, infrared radiation had a more pronounced influence on the drying curve, which was consistent with the observed temperature increase. As temperatures rise, molecular activity intensifies, and surface tension forces cause more water molecules to detach from the product matrix, effectively enhancing the effective diffusion coefficient of moisture [36]. Cherono et al. [37] also reported that samples dried using infrared heaters exhibit higher drying temperatures and faster rates, potentially triggering protein hydrolysis reactions, leading to loose muscle fiber structures and reduced water holding capacity. In this study, the drying method combining CU and IR shortened the drying time by 36.84 %, which was benefited from the synergistic effect between the two technologies.

**Fig.4:**
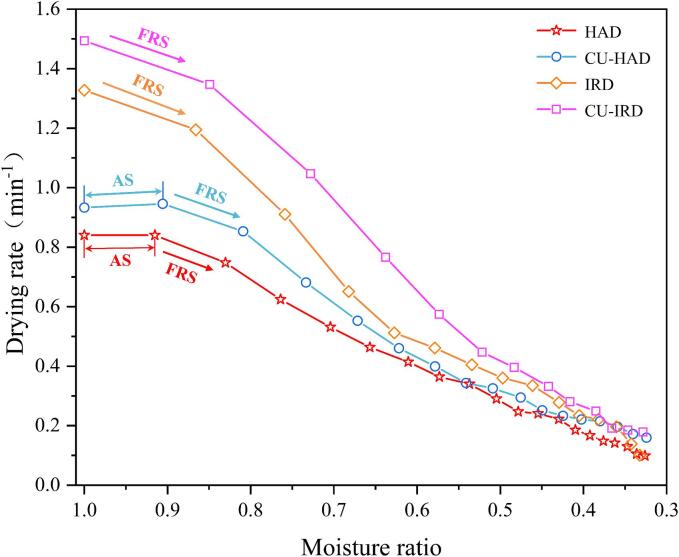
Drying rate of beef under different drying conditions. AS: accelerating stage, FRS: falling-rate stage.

Fig. 4 illustrates the relationship between the drying rate and moisture ratio under different drying conditions for beef. According to classical categorization of drying kinetics, the entire process generally consists of three stages: the heating-up (accelerating) stage, constant-rate stage, and falling-rate stage [38]. In our research, the drying rate of beef consistently decreased with decreasing moisture ratio, indicating that the falling-rate drying phase dominated the process. Notably, within the moisture ratio range of 0.9–1.0, both HAD and CU-HAD groups exhibited a brief accelerating period, which can be attributed to the relatively low heat flux density provided by hot air. Due to the limited heat flux density from hot air, a substantial portion of the initial input energy was primarily utilized to elevate the temperature of the material itself. Consequently, insufficient energy was available for water vaporization at the onset, resulting in a low initial rate of evaporation, thus giving the appearance of a transient accelerating stage [39].

Throughout the drying cycle, the use of IR significantly increased the drying rate during the initial half of the process, but a precipitous decline in the drying rate was observed in the latter stages. This may be attributed to the formation of a hard crust on the surface of the beef that impeded further moisture release at later stages. Parrouffe et al. [40] posited that IR ceased to be effective after a certain point (during the falling-rate period) due to low surface moisture content, which typically resulted in shallow penetration depths or reduced absorption rates. The application of CU, on the other hand, enabled an overall increase in the drying rate throughout the drying process, particularly during stages when moisture content was high. In the final stages of drying, ultrasound processing still maintained a relatively steady drying rate, effectively mitigating the downward trend in the rate curve, consistent with findings reported by Baeghbali et al. [41]. Regarding the former mechanism, it can be elucidated through the cavitation effect of ultrasound. When ultrasonic waves propagate within a fluid medium, they induce cyclic variations in local pressure, which consequently trigger the formation and violent implosion of cavitation bubbles [42]. As the fluid medium (moisture) diminishes over time, both the local pressure differential and the quantity of cavitation bubbles are correspondingly affected, resulting in a lessened micro-agitation and micro-jetting action caused by bubble collapse [43]. However, another significant role of ultrasound is its capacity to generate a series of alternating compression and expansion within the sample, thereby creating microchannel structures within the solid phase that facilitate fluid movement. These microchannels effectively improve mass transfer resistance during the latter stages of drying, thus expediting moisture evaporation efficiently [44].Gamboa-Santos et al. [45] have also substantiated the favorable impact of ultrasound treatment on enhancing mass transfer properties during the later phases of food drying in their research.

### 3.3 Drying model

Theoretical and empirical models were employed to fit experimental data obtained from four distinct drying methods in order to describe the drying process of beef mathematically, which was summarized in Table 2. The drying model deemed as the most suitable is the one with higher R^2^ and lower RMSE and SSE values [46].

All selected models effectively replicated the measured data, exhibiting R^2^ values ranging from 0.90 to 1.00. In this study, fitting the same model across different drying methods resulted in relatively minor differences in statistical parameters, which may be attributed to the inherent characteristics of the drying material itself. Except for the Newton (Lewis) model, other empirical models showed superior statistical metrics compared to Fick's second law, suggesting a certain limitation in the theoretical model when predicting the variation trend of MR over time. Among all drying methods, both the two-term and Approximation of diffusion models exhibited the highest R^2^ values and the lowest RESM and SSE values, thus being recognized as having exceptional descriptive capabilities for the drying kinetics of air-dried beef.

The effective moisture diffusion coefficient (D_eff_), a key parameter that measures the ability of internal moisture diffusion within a sample, is closely related to the drying rate and migration of moisture during the drying process [47]. In this study, the D_eff_ for the HAD, CU-HAD, IRD, and CU-IRD groups were found to be 4.188 × 10^-8^, 4.758 × 10^-8^, 5.083 × 10^-8^, 5.959 × 10^-8^ m^2^/min, respectively, consistent with the observed patterns in their corresponding drying kinetic curves. With the introduction of infrared radiation and ultrasound, the D_eff_ of beef increased by 42.29 % compared with HAD group, leading to a noticeable enhancement in drying efficiency.

### 3.4 Energy consumption (EC)

In various drying technologies, minimizing energy consumption remains a noteworthy challenge. In this study, energy consumption indicates the total energy expended by the laboratory-scale combined dryer in reducing the moisture content of beef from an initial level of 74.82 % (w. b.) to a final level of 50 % (w. b.). Table 3 presents the energy consumption values under different drying conditions for air-dried beef. Under different drying conditions, the energy consumption was recorded between 2.036 and 2.883 kWh. Evidently, the drying conditions significantly influence the energy consumption values (P ＜ 0.05), with the HAD group demonstrating the lowest energy expenditure of 2.036 kWh, which is closely related to the cost-effective attribute of hot air drying. Comparatively, IRD reduces the overall drying time with a modest increase of 9.33 % in energy consumption relative to HAD.

**Table 3:** Effect of different drying conditions on energy consumption and texture of air-dried meat.

Treatment	EC (kW·h)	Hardness (g)	Springiness	Cohesiveness	Chewiness (N)
HAD	2.036 ± 0.081^c^	2029.912 ± 63.875^a^	0.560 ± 0.008^a^	0.512 ± 0.010^a^	580.699 ± 11.390^a^
CU-HAD	2.813 ± 0.065^a^	1723.507 ± 182.718^b^	0.500 ± 0.016^b^	0.446 ± 0.008^b^	383.105 ± 41.872^b^
IRD	2.226 ± 0.042^b^	1379.989 ± 46.395^c^	0.527 ± 0.029^ab^	0.502 ± 0.033^a^	362.929 ± 15.280^bc^
CU-IRD	2.883 ± 0.082^a^	1189.153 ± 43.986^c^	0.539 ± 0.008^a^	0.497 ± 0.005^a^	317.889 ± 13.289^c^

Values followed by different letters in each column indicated significant differences (P < 0.05).

With respect to time benefits, the CU-HAD group exhibited a greater dependence on energy. While contact ultrasound and intermittent release modes have been reported to play critical roles in reducing transmission losses and enhancing energy efficiency [48], no significant advantages were observed in this study. Ozuna et al. [49] found that the texture of the material affects the efficacy of ultrasound. The softer and more porous structures show lower acoustic impedance. Beef's inherently elastic tissue structure and increased surface hardness during the drying process lead to attenuation in ultrasound propagation efficiency and cavitation effects. In addition, the difference in equipment efficiency (especially ultrasound and hot air devices) may also lead to this result.

In summary, the integration of ultrasound and infrared radiation into hot air drying undoubtedly contributed to time savings and enhanced production efficiency. Nevertheless, it concurrently increased energy consumption by 41.60 % compared to the HAD group. Relatively speaking, the IRD group demonstrated a more economically feasible energy performance, while the CU-HAD group held higher energy costs. Therefore, when optimizing drying processes, it is essential to strike a balance between time and energy cost considerations to identify the optimal trade-off point.

### 3.5 Low field nuclear magnetic resonance relaxation

Water is the main constituent of beef, accounting for about 75 % of the total weight. Based on the varying interactions and strength differences between water molecules and non-aqueous substances, the T_2b_ relaxation time (1–10 ms) is designated to represent water tightly bound to proteins (bound water), T_21_ relaxation time (10–100 ms) corresponds to water located within the myofibrils, sarcomeres, and cellular membrane gaps (immobilized water), while T_22_ relaxation time (100–1000 ms) pertains to freely mobile water within the extracellular spaces (free water) [50]. Prior research has established that the relaxation times, amplitudes, or areas of T_2_ populations are closely related to the internal structure or protein properties of fresh meat [51].

**Fig.5:**
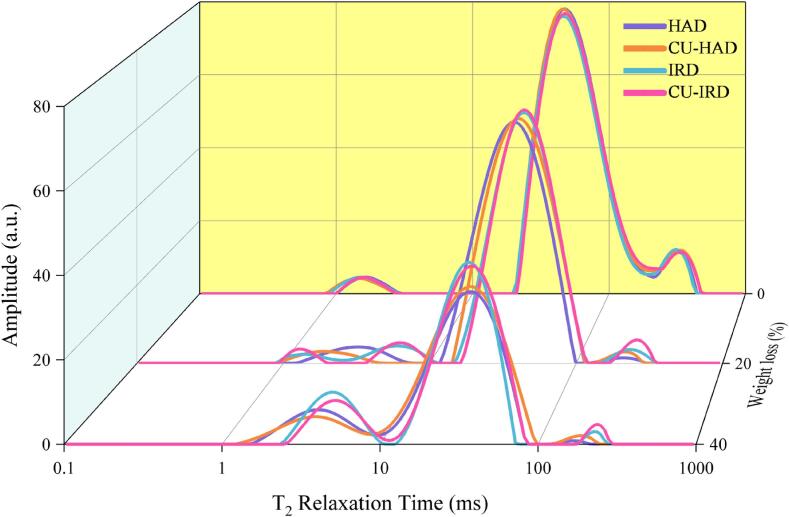
Effect of treatments on moisture distribution during beef drying.

Fig. 5 presents the T_2_ relaxation distributions of beef during different air-drying processes. At all measurement points, three distinct water populations centered around approximately 1–10 ms (T_2b_), 30–50 ms (T_21_), and 150–400 ms (T_22_) can be discerned. As the drying process advances, there is a clear trend of T_21_ and T_22_ relaxation times shifting toward lower relaxation times, coupled with a significant decrease (P < 0.05) in the corresponding integral areas, reflecting the gradual depletion of immobilized water within the muscle fiber structure and free water in the extracellular spaces. Micklander et al. [52] also reported similar findings and hypothesized that this phenomenon could arise from denaturation of myosin, leading to a reduction in the lattice spacing within muscle fibers. Correspondingly, Rao et al. [20] highlighted in their report that during air-drying of beef, denaturation of the myosin head led to enhanced hydrophobicity and decreased solubility of myofilament proteins. This effect may weaken the interaction forces between myofilament proteins and water molecules, facilitating the transition of immobilized water within the meat into a free water state.

In contrast, as the drying process continues, T_2b_ relaxation times and their corresponding peak areas A_2b_ display a progressive increase. Notably, the population centered around 1–10 ms (T_2b_) widens significantly, leading to an overlap of the immobilized water peak with the bound water peak. Han et al. [53] revealed that the heterogeneity of the sample structure increased, which amplified the heterogeneity of the environment surrounding water protons, hence causing a broader T_2_ distribution during heating. In addition, Li et al. [54] also reported that with the development of the drying process, most of the original high degree of freedom of immobile water was converted to free water and evaporated, and the remaining part formed a weak binding with tissue proteins, which is called “newly bound water” or “weakly-bound water”. It exhibits a relatively lower binding strength and longer T_2b_ relaxation times, primarily residing outside the immediate vicinity of solute surfaces (in the second hydration shell) [55]. In the present study, bound water peak split into a strongly-bound and a weakly-bound water peak when the moisture content drops to 69 % in the IRD and CU-IRD groups, eventually trending towards recombination.

Consistent with the findings reported by Krishnamurthy et al. [56], infrared radiation promoted the transformation of immobilized water to free water during drying, resulting in a rightward shift of T_21_ and T_22_ relaxation time. Parallel to this, the rate of moisture expulsion between muscle fiber bundles increased with rising temperatures. Furthermore, the high thermal effect and penetrating capability of far-infrared radiation caused rapid vibrations of water molecules within the beef, further weakening the bonding between myofilaments and water molecules, thus enhancing the mobility of water molecules [57].

Previous research has shown that exposing meat directly to ultrasound without brine enhanced tenderness but the retention of intramuscular constituents and water-holding capacity droped [58]. In the present study, the A_23_ peak area of the ultrasonically treated beef was significantly higher than that of the HAD groups, while the A_22_ peak area was decreased. This is due to the alternating positive and negative pressures generated by ultrasound, causing cell rupture and increased interstitial spaces between muscle fibers. Some of the immobilized water previously trapped within the muscle fibers becomes destabilized and leaks out, thereby increasing the overall water mobility [59]. Pearce et al. [60] also noted that an increase in T_21_ time signifies an increase in the internal space within muscle fibers, while an increase in T_22_ time represents expanded spaces in the outer regions of muscle fibers, which corresponds to the unique mechanical action of ultrasound on muscle tissue.

**Fig.6:**
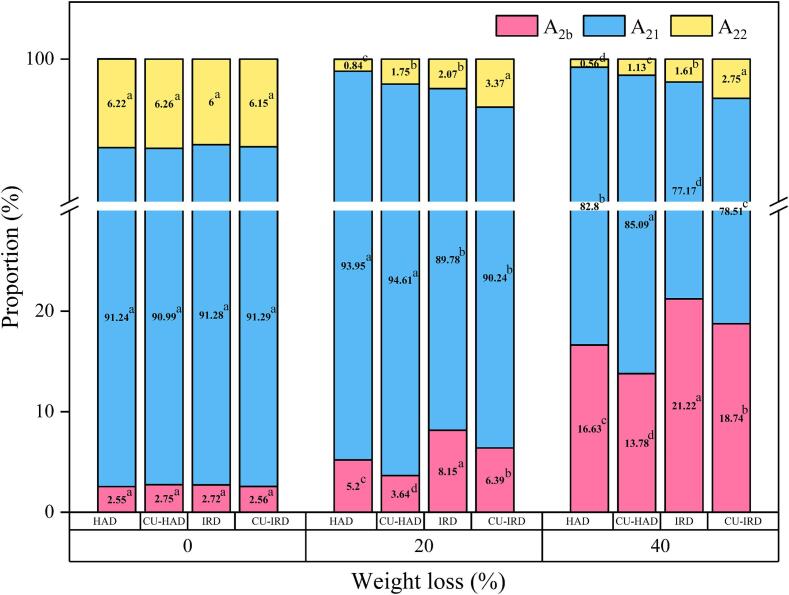
Effect of different drying treatments on the proportion of T_2_ peak area (mean ± SD, n = 3). Values followed by different letters in the same weight loss and component indicated significant differences (P < 0.05).

Fig. 6 depicts the corresponding fractions (P_2b_, P_21_, and P_22_) of T_2_ distributions under different drying conditions. Throughout the drying process, significant ascending and descending trends were observed in the P_2b_ and P_22_ fractions, respectively. Conversely, P_21_ remained relatively stable in the early stages (ranging from 89.78 % to 94.61 %), even showing a slight increase in the HAD and CU-HAD groups. This phenomenon may be attributed to the relatively high availability of free water and the low degree of myofibrillar denaturation during the initial stages of drying, which restricts the conversion of immobilized water to other forms of water molecules. And this process is particularly sensitive to temperature fluctuations.

Infrared radiation significantly augmented the peak proportion (8.15 and 21.22 % peak percentage at 20 % and 40 % mass loss, respectively), which can be rationalized by the thermal-induced gelation mechanism of myosin. When the temperature exceeds 40 °C, the gel structure formed by myosin effectively confines the mobility of immobilized water within the myofibrils, prompting its transition to the low relaxation time segment, i.e., bound water [61]. However, the increase in bound water fraction may hinder the efficiency of converting immobilized water to free water, potentially leading to a decline in drying efficiency of later stages. Addressing this issue, the incorporation of ultrasound provided some relief. Previous research has indicated that ultrasound-generated cavitation effects can induce adjustments in the intermolecular forces and internal conformations of myofibrillar proteins (MPs), thereby altering their secondary structures and associated functional properties [62]. Yao et al. [63] reported that ultrasound treatment facilitated the exposure of hydrophobic amino acid residues within proteins, thereby reducing their hydration capacity.

### 3.6 Thermal behaviour

Severe protein denaturation has been shown to impair the protein's affinity for water, leading to a reduction in its water-holding capacity [64]. Differential scanning calorimetry (DSC) stands as a potent analytical technique for elucidating the thermodynamic transitions of proteins in muscle tissue and extracted proteins. Specifically, the peak denaturation temperature (T_max_), which denotes the point of highest heat absorption rate, is customarily utilized to classify the denaturation of different proteins [65]. Meanwhile, the denaturation enthalpy (ΔH) serves as a pivotal parameter in characterizing the denaturation profile, offering insights into the energetic changes accompanying the denaturation event.

The parameters of protein denaturation are given in Table 4. The investigation revealed that beef samples subjected to differential treatments exhibited three prominent endothermic peaks at approximately 58–59 °C, 69–74 °C, and 81–83 °C, which represented the thermal denaturation process of myosin, sarcoplasmic protein and actin respectively [66]. Collectively, the ΔH values of the dried samples exhibited a notable decline, especially ΔH_1_ and ΔH_2_, which are more susceptible to thermal processing. This implicated a compromise in the thermal stability of the proteins during the dehydration procedure. Aktaş et al. [67] suggested this observation could be linked to the loss of moisture during drying, as the presence of water can inhibit myofibrillar protein denaturation, potentially through limiting hydrogen bond cleavage or mitigating the disruptive effect of ionic strength on protein conformation. Additionally, the denaturation temperatures of these endothermic peaks were observed to escalate under harsher treatment regimens, which may be related to the increase of crosslinking degree of protein gels induced by heat [68].

**Table 4:** Thermal denaturation temperature and enthalpy of protein with different treatments.

Samples	Tmax_1_(°C)	ΔH_1_(J/g)	Tmax_2_(°C)	ΔH_2_(J/g)	Tmax_3_(°C)	ΔH_3_(J/g)
CT	58.73 ± 0.66^b^	0.485 ± 0.054^a^	69.67 ± 0.55^d^	0.502 ± 0.070^a^	81.42 ± 0.72^b^	0.595 ± 0.049^a^
HAD	58.81 ± 0.13^b^	0.447 ± 0.068^a^	70.23 ± 0.42^d^	0.472 ± 0.082^ab^	81.61 ± 0.72^b^	0.555 ± 0.043^a^
CU-HAD	59.73 ± 0.24^a^	0.318 ± 0.086^b^	71.16 ± 0.19^c^	0.485 ± 0.038^ab^	82.61 ± 0.73^ab^	0.562 ± 0.047^a^
IRD	/	/	73.01 ± 0.31^b^	0.423 ± 0.038^ab^	83.26 ± 0.73^a^	0.533 ± 0.066^a^
CU-IRD	/	/	74.05 ± 0.67^a^	0.385 ± 0.025^b^	83.60 ± 0.71^a^	0.518 ± 0.097^a^

Values followed by different letters in each column indicated significant differences (P < 0.05). CT: fresh samples. HAD: hot air drying, CU-HAD: hot air drying with CU, IRD: hot air drying with IR, CU-IRD: hot air drying with CU and IR.

The HAD group displayed similar curve to the CT group, with no statistically significant differences (P > 0.05) observed in the temperatures of endothermic peaks (58.81 and 58.73 °C) or enthalpy values (0.447 and 0.485 J/g), suggesting that the HAD group maintained the intact protein structure. Intriguingly, despite the recorded maximum temperatures during the drying process (depicted in Fig. 2) being significantly lower than the denaturation temperature of myosin, the disappearance of the first endothermic peak was noted in both IRD and CU-IRD groups. This finding implied that myosin denaturation was not solely an instantaneous event triggered upon reaching a temperature threshold. Complete myosin denaturation under these conditions might stem from multiple factors, including direct absorption of IR energy by protein functional groups and the consequent breakdown of covalent bonds [27]. As previously mentioned, the infrared absorption spectrum of beef (Fig. S1) registers sensitivity to several functional groups (O-H, C-H, C = O, N-C = O, and N-H) towards infrared radiation. Based on the bond energy of covalent bonds and the magnitude of infrared absorption peaks, it can be inferred that the O-H, C-H, N-C = O, and N-H groups were more prone to break during this process, consequently resulting in the disappearance of thermal peaks of myosin.

Moreover, a significant decrease in ΔH_1_ (from 0.485 to 0.318 J/g)was detected in the CU-HAD group (P < 0.05), indicating heightened myosin instability as ultrasonic exposure time increased. Ultrasonic cavitation intensified protein extraction through mechanisms such as bubble implosion, micro-mechanical shock waves, and micro-jetting, which potentially weaken protein structures and induce their dissociation [69].

### 3.7 Surface hydrophobicity

**Fig.7:**
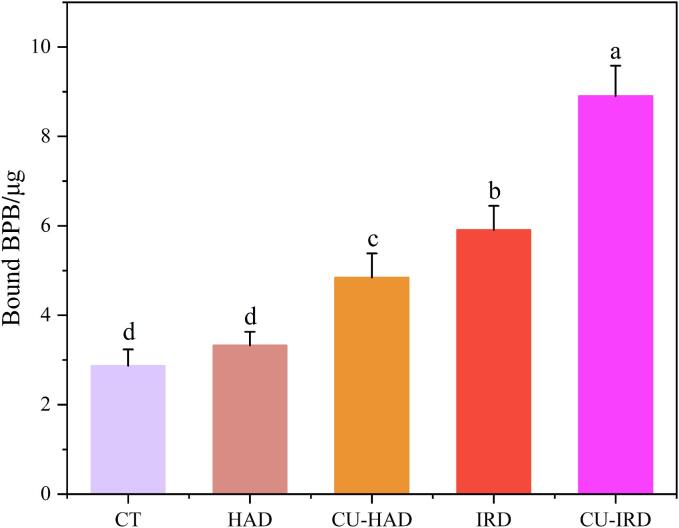
Protein surface hydrophobicity after various drying treatments.

Protein surface hydrophobicity is often used to characterize the number of hydrophobic amino acid residues on the surface of protein molecule and plays a central role in determining the stability, spatial conformation of proteins and their biological functions [70]. Fig. 7 illustrates the variations in protein surface hydrophobicity following different drying treatments. Relative to the CT group (2.87 μg), the HAD group (3.32 μg) displayed only a marginal increase in protein hydrophobicity (P > 0.05), whereas all other groups exhibited significant increases (P < 0.05), ranging from 4.84 μ g to 8.90 μ g. Consistent with the report of Chelh et al. [71], muscle fiber proteins remained stable at 30 °C, while elevated temperatures lead to the exposure of aromatic and long-chain aliphatic amino acids, particularly in myosin. In this study, the exposure of hydrophobic groups on the protein surface and the reduction in denaturation enthalpy collectively suggested a higher degree of protein denaturation. We speculated that this was due to the disruption of the forces used to maintain protein stability, such as hydrogen bonds and ionic interactions, which were sensitive to thermal processing. The molecular vibrations and friction induced by infrared radiation also affected the microstructure and covalent bonds (Fig. S1), resulting in more pronounced changes in surface hydrophobicity [72]. Additionally, the oxidative modifications triggered by cavitation effect might contribute to an extra increment in protein surface hydrophobicity. Kang et al. [73] reported that prolonged ultrasound treatment led to the near-complete exposure of buried hydrophobic regions onto the protein surface or into a more polar surrounding environment.

Furthermore, this study revealed a negative correlation between P_21_ and protein surface hydrophobicity, whereas P_22_ correlated positively (Fig. S2). These findings aligned with those of Song et al. [74], who demonstrated that increased protein surface hydrophobicity was associated with a decline in water-holding capacity, thereby explaining enhanced intramuscular water mobility and the conversion of bound to free water.

### 3.8 Nuclear magnetic resonance imaging (NMRI)

**Fig.8:**
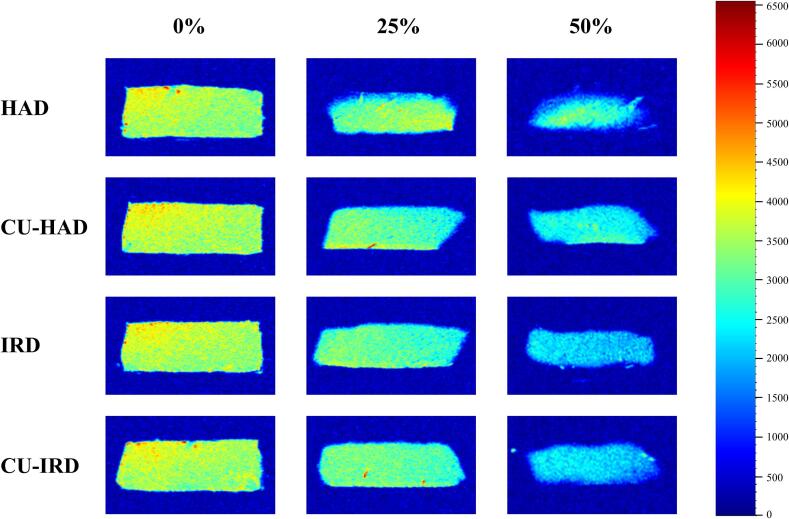
2D H-proton density images of beef during different drying processes. 0%, 25%, and 50% denote the amount of weight lost.

The MRI images of beef during different drying processes are presented in Fig. 8. MRI signal brightness is positively correlated with the concentration of hydrogen protons within the sample. As drying progresses, the brightness of the H-proton density images gradually decreased, which may be attributed to the conversion of immobilized water within the beef to free water and then dissipated into the drying environment [75]. Concurrently, uneven shrinkage and deformation occurred across various parts of the sample, primarily driven by the combined effects of thermal and mechanical forces.

In the HAD group, the signal intensity at the upper surface and side edges of the samples was significantly lower than in the interior and bottom regions, accompanied by visibly shrinkage. This was primarily because hot air only effectively removed moisture from the surface and sides of the sample, resulting in the formation of a hard crust on the surface. The uneven distribution of temperature within the sample hindered efficient moisture transport, leading to a large moisture gradient inside the sample. During the HAD process, the rate at which moisture migrates from the interior to the surface lagged behind the evaporation rate of surface moisture due to the weaker thermal effect, culminating in diverse physical changes such as hardening and shrinkage at the surface, which is consistent with the description of Li et al. [18].

Compared to other drying methods, IRD group possessed a higher heat transfer efficiency and a certain degree of penetration capacity [76], enabling the creation of a relatively uniform temperature distribution within the material. This uniformly heated environment, coupled with enhanced vibration of water molecules, facilitated the mobilization of both immobilized and free water, ensuring prompt replenishment of surface moisture. Consequently, the moisture gradient characteristics and the extent of shrinkage are markedly alleviated in IRD group.

In the case of ultrasound-treated beef samples, the distribution of H-proton signals exhibits a higher degree of homogeneity. The underlying mechanisms behind this observation include the sustained application of ultrasound that induces a sponge-like effect and the formation of micro-channels [42]. Additionally, the wrinkles and lateral stretching observed on the surface of beef samples might result from the alternating positive and negative pressure generated by the ultrasound, leading to Z-line disruptions, secondary structural changes in myofibrillar proteins, and myosin denaturation [77], [78].

Shi et al. [79] reported that the combined application of far-infrared and ultrasound reduced structural shrinkage in banana samples effectively, increase porosity, and generate more and larger-sized diffusion micro-channels, thereby expediting the migration of moisture within bananas. In the present study, the CU-IRD group displayed a more uniform moisture distribution along with reduced shrinkage rates, and a noticeable improvement in the wrinkling phenomenon compared to the CU-HAD group.

### 3.9 Texture

Texture is a sensory attribute that refers to the tactile response generated when food interacts with oral structures. It encompasses a series of physical parameters based on tissue structure and is regarded as one of the most critical quality attributes of food products. During thermal processing, varying degrees of myofibrillar contraction and denaturation of collagen contribute to the distinctive textural properties of the food [80]. Table 3 records the TPA parameters for beef samples subjected to different drying treatments.

Hardness is the force required to change the shape of an object, while chewiness reflects the energy expended in grinding solid food into a swallowable state. Research has established that both these attributes are inherently related to beef tenderness, often exhibiting similar trend changes [81]. In this study, significant differences (P < 0.05) were observed in the hardness and chewiness of beef samples subjected to various drying treatments, primarily due to the surface hardening phenomenon occurring during the drying process. As beef samples were exposed to hot air over extended periods, surface moisture continuously evaporated, causing proteins, fats, and connective tissues to adhere and form rigid structures, thereby increasing resistance to external forces. Relative to the HAD group, all other drying methods significantly reduced the hardness and chewiness of the air-dried beef (P < 0.05). Among them, the CU-IRD group presented the lowest hardness (1189.153 g) and chewability (317.889 N), which were 40.42 % and 45.25 % lower than HAD respectively. This reduction can largely be attributed to the accelerated internal-to-surface moisture transfer rates facilitated by IR and CU technologies, which partially alleviated the mismatch in moisture transportation rates within and outside the beef and the excessive shrinkage.

Springiness refers to an object's ability to return to its original state after deformation following the removal of an external force. It was observed that, compared to the HAD group (0.560), CU-HAD treatment (0.539) had a significantly negative effect on springiness (P < 0.05), with some decline also noted in other groups but not reaching statistical significance (P > 0.05). The prolonged ultrasound treatment in the CU-HAD group likely caused noticeable changes to the muscle fiber structure. Previous report has linked springiness to the mechanical properties of connective tissue, particularly the thermal denaturation and crosslinking degree of collagen. Wang et al. [82] found that applying 300 W ultrasonication led to a significant decrease in the peak temperature of the endotherm (T_m_) of collagen in beef samples, speculating that the physical effect of ultrasound might alter the hydration of collagen's triple helix structure and intermolecular crosslink status. Chang et al. [83] further corroborated this notion, reporting that longer ultrasound exposure times resulted in increasingly evident granulation and denaturation of collagen fibers. The cavitation-induced breakdown, depolymerization, and dissolution of collagen fibers weakened the mechanical strength and resistance of the tissue, ultimately decreasing springiness.

Cohesiveness signifies the magnitude of internal binding forces needed to maintain the integrity of a food's structure and typically relates to the completeness and restorative capacity of the meat's tissue structure. In this study, cohesiveness exhibited a trend similar to elasticity. We hypothesize that the integrity of muscle fibers in the CU-HAD group samples were damaged. Prolonged ultrasonic treatment could lead to muscle cell rupture, increased extracellular spaces, and intracellular vacuolation [58]. Moreover, the decreased thermal stability of collagen and loss of muscle fiber integrity may render the collagen network looser and more disorganized. The expansion of internal voids and irreversible damage to the collagen network reduce the overall cohesiveness and tensile strength [84].

### 3.10 Microstructure

**Fig.9:**
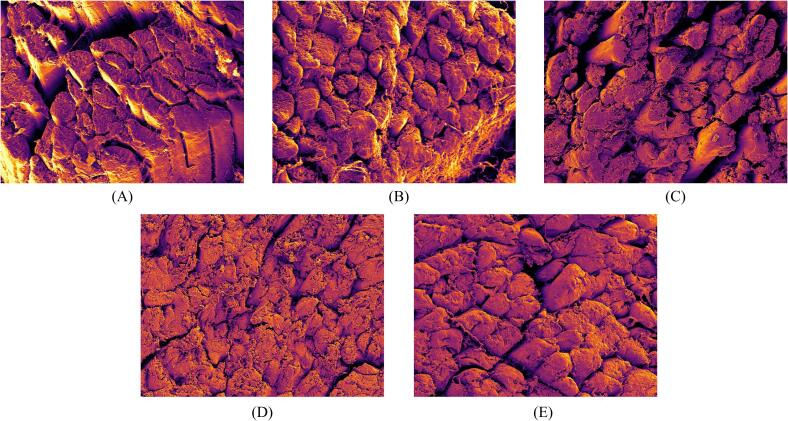
Changes of beef microstructure after different drying conditions. Different capital letters (A-E) represent CT, HAD, CU-HAD, IRD and CU-IRD groups, respectively.

Microstructure serves as a crucial indicator for explaining the textural changes in dried meat products. Fig. 9 depicts the microscopic structure of samples under a 500x magnification, allowing clear distinction of variations in muscle fiber structure and porosity. The elliptical muscle fibers in the fresh samples were disorganized with distinct gaps between fiber bundles, which may be related to the freeze–thaw cycles experienced by the raw material. Ice crystal formation and melting may cause muscle fiber disintegration and connective tissue damage, leading to substantial mechanical injury and a loose structure [85]. Additionally, the intramuscular connective tissue in fresh samples was clearly discernible and intact, appearing as thicker layers surrounding muscle fiber bundles (perimysium) and thinner layers enveloping individual muscle fibers (endomysium).

Upon single hot air drying, thawed meat samples show significantly reduced spacing between muscle fibers, resulting in a denser tissue structure and inferior overall texture, consistent with previous report [86]. Undoubtedly, the uneven heating from hot air exacerbated the nonuniformity of muscle fiber morphology in the HAD and CU-HAD groups, evidenced by significant disparities between inner and peripheral diameters. However, it is noteworthy that milder drying conditions relatively mitigated structural damage to some extent, allowing the HAD group samples to retain relatively intact fiber structures.

Consistent with the study by Chang et al. [58], ultrasonically treated samples exhibited partial disruption of the muscle membranes, leading to loss of fiber integrity and branching of myofibrils. With increasing ultrasound treatment duration, the thickness and content of perimysium and endomysium gradually decreased. This was attributed to the disruptive effect of ultrasound cavitation on the crosslinking state and stability of collagen. Previous study has shown that the tenderness of meat is directly related to the level of connective tissue, and the tenderizing effect of ultrasound on meat is due to the increased solubility of collagen in the connective tissue [82]. Yang et al. [87] reported that ultrasound creates microchannels conducive to water migration and flow through bubble explosion and high-frequency oscillation, further weakening tissue shrinkage. This phenomenon was more pronounced in the CU-HAD group, where a looser structure and visibly fractured fibers visually confirmed the improved hardness and chewability of the air-dried meat post-ultrasound treatment.

With increasing infrared heating time, muscle fiber structures began to disintegrate, and collagen transitioned from a gel-like network to a granular state. This process was closely related to the temperature changes and duration of infrared heating, as its high-temperature effect accelerated the thermolysis of myofibrillar proteins and the shrinkage of collagen. Li et al. [88] pointed out in their report that environments above 40 °C favor the accumulation of thermally denatured myofibrillar components, particularly denatured myosin. Wattanachant et al. [89] also emphasized that as samples absorb more heat, the gel network formed by intramuscular connective tissue (IMCT) collagen, thermally denatured and aggregated myofibrillar proteins, and sarcoplasmic proteins becomes unstable. Notably, CH-IRD group exhibited better fiber diameter and fiber gap than the IRD group, indicating that a higher degree of protein denaturation does not necessarily equate to better textural properties in air-dried meat products.

## 4 Conclusions

A novel drying technology combining contact ultrasound (CU) with infrared radiation (IR) was introduced, and moisture migration patterns and sample characteristics under four distinct drying modes were examind. The findings revealed that the synergistic action of CU and IR markedly accelerates mass transfer rates during drying, primarily attributed to reduced myosin thermal denaturation enthalpy and increased exposure of hydrophobic groups on protein surfaces, which in turn diminishes the water-holding capacity of muscle fibers. The sponge effect of ultrasound reduced the gradient phenomenon during the drying process and changed the fiber structure, which contributes to the texture of the product. By considering moisture distribution and texture, this study demonstrated that the drying system combining CU with IR enhanced efficiency while effectively mitigating non-uniformity and tenderness loss associated with drying. From energy consumption perspective, the ultrasound system has not shown significant advantages. Therefore, it is necessary to optimize the structure of ultrasoud equipment in order to improve its energy efficiency.

## CRediT authorship contribution statement

**Jiahua Gao:** Writing – review & editing, Writing – original draft, Formal analysis, Data curation. **Siyu Cheng:** Formal analysis, Data curation. **Xianming Zeng:** Project administration. **Xiaomei Sun:** Investigation. **Yun Bai:** Project administration. **Songmei Hu:** Funding acquisition. **Jianping Yue:** Project administration, Funding acquisition. **Xiaobo Yu:** Project administration. **Minwei Zhang:** Data curation. **Xinglian Xu:** Project administration. **Minyi Han:** Writing – review & editing, Project administration.

## Declaration of competing interest

The authors declare that they have no known competing financial interests or personal relationships that could have appeared to influence the work reported in this paper.

## References

[1] Aykın D.E. (2023). Dried meat products obtained by different methods from past to present[J]. Food Rev. Intl..

[2] Jiang L., Chen Y., Deng L. (2022). Bacterial community diversity and its potential contributions to the flavor components of traditional smoked horsemeat sausage in Xinjiang, China[J]. Front. Microbiol..

[3] Rao W., Wang Z., Li G. (2020). Formation of crust of dried meat and its relationship to moisture migration during air drying[J]. J. Food Process. Preserv..

[4] Ren Y., Sun D.-W. (2022). Monitoring of moisture contents and rehydration rates of microwave vacuum and hot air dehydrated beef slices and splits using hyperspectral imaging[J]. Food Chem..

[5] Doymaz İ., Karasu S., Baslar M. (2016). Effects of infrared heating on drying kinetics, antioxidant activity, phenolic content, and color of jujube fruit[J]. J. Food Meas. Charact..

[6] Obajemihi O.I., Cheng J.-H., Sun D.-W. (2023). Novel sequential and simultaneous infrared-accelerated drying technologies for the food industry: Principles, applications and challenges[J]. Crit. Rev. Food Sci. Nutr..

[7] Jain D., Pathare P.B. (2004). Selection and evaluation of thin layer drying models for infrared radiative and convective drying of onion slices[J]. Biosyst. Eng..

[8] Sakai N., Hanzawa T. (1994). Applications and advances in far-infrared heating in Japan[J]. Trends Food Sci. Technol..

[9] Garcia-Perez J.V., Carcel J.A., Simal S. (2013). Ultrasonic Intensification of Grape Stalk Convective Drying: Kinetic and Energy Efficiency[J]. Drying Technol..

[10] Tao Y., Sun D.-W. (2015). Enhancement of food processes by ultrasound: A review[J]. Crit. Rev. Food Sci. Nutr..

[11] Sabarez H.T., Gallego-Juarez J.A., Riera E. (2012). Ultrasonic-Assisted Convective Drying of Apple Slices[J]. Drying Technol..

[12] Cárcel J.A., García-Pérez J.V., Benedito J. (2012). Food process innovation through new technologies: Use of ultrasound[J]. J. Food Eng..

[13] Nowacka M., Wiktor A., Śledź M. (2012). Drying of ultrasound pretreated apple and its selected physical properties[J]. J. Food Eng..

[14] Mason T.J., Lorimer J.P., Bates D.M. (1992). Quantifying sonochemistry: Casting some light on a ‘black art’[J]. Ultrasonics.

[15] Sánchez-Torres E.A., Abril B., Benedito J. (2022). Airborne ultrasonic application on hot air-drying of pork liver. Intensification of moisture transport and impact on protein solubility[J]. Ultrason. Sonochem..

[16] Gong C., Liao M., Zhang H. (2020). Investigation of Hot Air-Assisted Radio Frequency as a Final-Stage Drying of Pre-dried Carrot Cubes[J]. Food Bioproc. Tech..

[17] Ertekin C., Firat M.Z. (2017). A comprehensive review of thin-layer drying models used in agricultural products[J]. Crit. Rev. Food Sci. Nutr..

[18] Li X., Xie X., Zhang C. (2018). Role of mid- and far-infrared for improving dehydration efficiency in beef jerky drying[J]. Drying Technol..

[19] Zhang Y., Feng M., Zhang J. (2022). Peptidomics insights into the interplay between the pre-digestion effect of mixed starters and the digestive pattern of sausage proteins[J]. Food Res. Int..

[20] Rao W., Wang Z., Shen Q. (2018). LF-NMR to explore water migration and water–protein interaction of lamb meat being air-dried at 35°C[J]. Drying Technol..

[21] Sheridan P., Shilton N. (1999). Application of far infra-red radiation to cooking of meat products[J]. J. Food Eng..

[22] Hou T., Chen Y., Wang Z. (2018). Experimental study of fouling process and antifouling effect in convective heat transfer under ultrasonic treatment[J]. Appl. Therm. Eng..

[23] Zhang D., Jiang E., Zhou J. (2020). Investigation on enhanced mechanism of heat transfer assisted by ultrasonic vibration[J]. Int. Commun. Heat Mass Transfer.

[24] Musielak G., Mierzwa D. (2021). Enhancement of convection heat transfer in air using ultrasound[J]. Appl. Sci..

[25] Schössler K., Jäger H., Knorr D. (2012). Novel contact ultrasound system for the accelerated freeze-drying of vegetables[J]. Innov. Food Sci. Emerg. Technol..

[26] Gangidi R r., Proctor A, Pohlman F w. Rapid determination of spinal cord content in ground beef by attenuated total reflectance fourier transform infrared spectroscopy[J]. Journal of Food Science, 2003, 68(1): 124-127.

[27] Iizuka K., Aishima T. (1999). Tenderization of beef with pineapple juice monitored by fourier transform infrared spectroscopy and chemometric analysis[J]. J. Food Sci..

[28] Onwude D.I., Hashim N., Abdan K. (2019). The effectiveness of combined infrared and hot-air drying strategies for sweet potato[J]. J. Food Eng..

[29] Datta A.K., Ni H. (2002). Infrared and hot-air-assisted microwave heating of foods for control of surface moisture[J]. J. Food Eng..

[30] Zhang X.-L., Zhong C.-S., Mujumdar A.S. (2019). Cold plasma pretreatment enhances drying kinetics and quality attributes of chili pepper (*Capsicum annuum L.*)[J]. J. Food Eng..

[31] Bai J.-W., Sun D.-W., Xiao H.-W. (2013). Novel high-humidity hot air impingement blanching (HHAIB) pretreatment enhances drying kinetics and color attributes of seedless grapes[J]. Innov. Food Sci. Emerg. Technol..

[32] Baslar M., Kilicli M., Toker O.S. (2014). Ultrasonic vacuum drying technique as a novel process for shortening the drying period for beef and chicken meats[J]. Innov. Food Sci. Emerg. Technol..

[33] García-Pérez J.V., Rosselló C., Cárcel J.A. (2006). Effect of air temperature on convective drying assisted by high power ultrasound[J]. Defect and Diffusion Forum.

[34] Charoux C.M.G., Ojha K.S., O’Donnell C.P. (2017). Applications of airborne ultrasonic technology in the food industry[J]. J. Food Eng..

[35] Tekin Z.H., Baslar M. (2018). The effect of ultrasound-assisted vacuum drying on the drying rate and quality of red peppers[J]. J. Therm. Anal. Calorim..

[36] Taghinezhad E., Kaveh M., Szumny A. (2021). Optimization and prediction of the drying and quality of turnip slices by convective-infrared dryer under various pretreatments by RSM and ANFIS methods[J]. Foods.

[37] Cherono K., Mwithiga G., Schmidt S. (2016). Infrared drying as a potential alternative to convective drying for biltong production[J]. Italian Journal of Food Safety.

[38] Prachayawarakorn S. (2019). Drying technologies for foods: fundamentals and applications[J]. Drying Technol..

[39] McMinn W.A.M., Magee T.R.A. (1999). Principles, methods and applications of the convective drying of foodstuffs[J]. Food Bioprod. Process..

[40] Parrouffe J.M., Dostie M., Navarri P. (1997). Heat and mass transfer relationship in combined infrared and convective drying[J]. Drying Technol..

[41] Baeghbali V., Ngadi M., Niakousari M. (2020). Effects of ultrasound and infrared assisted conductive hydro-drying, freeze-drying and oven drying on physicochemical properties of okra slices[J]. Innovative Food Science & Emerging Technologies.

[42] Huang D., Men K., Li D. (2020). Application of ultrasound technology in the drying of food products[J]. Ultrason. Sonochem..

[43] Arvanitoyannis I.S., Kotsanopoulos K.V., Savva A.G. (2017). Use of ultrasounds in the food industry–methods and effects on quality, safety, and organoleptic characteristics of foods: A review[J]. Crit. Rev. Food Sci. Nutr..

[44] Soria A.C., Villamiel M. (2010). Effect of ultrasound on the technological properties and bioactivity of food: A review[J]. Trends Food Sci. Technol..

[45] Gamboa-Santos J., Montilla A., Cárcel J.A. (2014). Air-borne ultrasound application in the convective drying of strawberry[J]. J. Food Eng..

[46] Pardeshi I.L., Arora S., Borker P.A. (2009). Thin-layer drying of green peas and selection of a suitable thin-layer drying model[J]. Drying Technol..

[47] Wang Y., Zhang L., Johnson J. (2014). Developing hot air-assisted radio frequency drying for in-shell macadamia nuts[J]. Food Bioproc. Tech..

[48] Musielak G., Mierzwa D., Kroehnke J. (2016). Food drying enhancement by ultrasound – a review[J]. Trends Food Sci. Technol..

[49] Ozuna C., Gómez Álvarez-Arenas T., Riera E. (2014). Influence of material structure on air-borne ultrasonic application in drying[J]. Ultrason. Sonochem..

[50] Belton P.S., Jackson R.R., Packer K.J. (1972). Pulsed NMR studies of water in striated muscle: I. Transverse nuclear spin relaxation times and freezing effects[J]. Biochim. Biophys. Acta Gen. Subj..

[51] Straadt I.K., Rasmussen M., Andersen H.J. (2007). Aging-induced changes in microstructure and water distribution in fresh and cooked pork in relation to water-holding capacity and cooking loss – a combined confocal laser scanning microscopy (CLSM) and low-field nuclear magnetic resonance relaxation study[J]. Meat Sci..

[52] Micklander E., Peshlov B., Purslow P.P. (2002). NMR-cooking: monitoring the changes in meat during cooking by low-field 1H-NMR[J]. Trends Food Sci. Technol..

[53] Han M., Wang P., Xu X. (2014). Low-field NMR study of heat-induced gelation of pork myofibrillar proteins and its relationship with microstructural characteristics[J]. Food Res. Int..

[54] Li X., Ma L.Z., Tao Y. (2012). Low field-NMR in measuring water mobility and distribution in beef granules during drying process[J]. Adv. Mat. Res..

[55] Perticaroli S., Ehlers G., Stanley C.B. (2017). Description of hydration water in protein (green fluorescent protein) solution[J]. Journal of the American Chemical Society.

[56] Krishnamurthy K., Khurana H.K., Soojin J. (2008). Infrared heating in food processing: An overview[J]. Compr. Rev. Food Sci. Food Saf..

[57] Abbaspour-Gilandeh Y., Kaveh M., Fatemi H. (2021). Effect of pretreatments on convective and infrared drying kinetics, energy consumption and quality of terebinth[J]. Appl. Sci..

[58] Chang H.-J., Wang Q., Tang C.-H. (2015). Effects of ultrasound treatment on connective tissue collagen and meat quality of beef *semitendinosus* muscle[J]. J. Food Qual..

[59] Chen L., Chai Y., Luo J. (2021). Apoptotic changes and myofibrils degradation in post-mortem chicken muscles by ultrasonic processing[J]. LWT Food Sci. Technol..

[60] Pearce K.L., Rosenvold K., Andersen H.J. (2011). Water distribution and mobility in meat during the conversion of muscle to meat and ageing and the impacts on fresh meat quality attributes — a review[J]. Meat Sci..

[61] Bertram H.C., Kohler A., Böcker U. (2006). Heat-induced changes in myofibrillar protein structures and myowater of two pork qualities. A combined FT-IR spectroscopy and low-field NMR relaxometry study[J]. J. Agric. Food Chem..

[62] Khatkar A.B., Kaur A., Khatkar S.K. (2018). Characterization of heat-stable whey protein: Impact of ultrasound on rheological, thermal, structural and morphological properties[J]. Ultrasonics Sonochemistry.

[63] Yao K., Xia Y., Gao H. (2019). Influence of ultrasonic power and ultrasonic time on the physicochemical and functional properties of whey protein isolate[J]. Int. J. Food Eng..

[64] Deng Y., Rosenvold K., Karlsson A.H. (2002). Relationship between thermal denaturation of porcine muscle proteins and water-holding capacity[J]. J. Food Sci..

[65] Purslow P.P. (2018). Contribution of collagen and connective tissue to cooked meat toughness; some paradigms reviewed[J]. Meat Sci..

[66] Parsons S.E., Patterson R.L.S. (1986). Assessment of the previous heat treatment given to meat products in the temperature range 40°–90°C. Part 2: Differential scanning calorimetry, a preliminary study[J]. International Journal of Food Science & Technology.

[67] Aktaş N., Aksu M.I., Kaya M. (2005). Changes in myofibrillar proteins during processing of pastirma (Turkish dry meat product) produced with commercial starter cultures[J]. Food Chem..

[68] Shang S., Liu Y., Jiang P. (2023). Effects of partial replacement of unwashed Antarctic krill surimi by Litopenaeus vannamei surimi on the heat-induced gelling and three-dimensional-printing properties[J]. Journal of Texture Studies.

[69] Ojha K.S., Keenan D.F., Bright A. (2016). Ultrasound assisted diffusion of sodium salt replacer and effect on physicochemical properties of pork meat[J]. Int. J. Food Sci. Technol..

[70] Chandrapala J., Zisu B., Palmer M. (2011). Effects of ultrasound on the thermal and structural characteristics of proteins in reconstituted whey protein concentrate[J]. Ultrason. Sonochem..

[71] Chelh I., Gatellier P., Santé-Lhoutellier V. (2006). Technical note: A simplified procedure for myofibril hydrophobicity determination[J]. Meat Sci..

[72] Han Z., Cai M., Cheng J.-H. (2018). Effects of electric fields and electromagnetic wave on food protein structure and functionality: A review[J]. Trends Food Sci. Technol..

[73] Kang D., Zou Y., Cheng Y. (2016). Effects of power ultrasound on oxidation and structure of beef proteins during curing processing[J]. Ultrason. Sonochem..

[74] Song Y., Huang F., Li X. (2021). Effects of different wet heating methods on the water distribution, microstructure and protein denaturation of pork steaks[J]. Int. J. Food Sci. Technol..

[75] Ling J.-G., Xuan X.-T., Yu N. (2020). High pressure-assisted vacuum-freeze drying: A novel, efficient way to accelerate moisture migration in shrimp processing[J]. J. Food Sci..

[76] Zhang Y., Zhu G., Li X. (2020). Combined medium- and short-wave infrared and hot air impingement drying of sponge gourd (*Luffa cylindrical*) slices[J]. J. Food Eng..

[77] Kang D., Wang A., Zhou G. (2016). Power ultrasonic on mass transport of beef: Effects of ultrasound intensity and NaCl concentration[J]. Innov. Food Sci. Emerg. Technol..

[78] Xue S., Xu X., Shan H. (2018). Effects of high-intensity ultrasound, high-pressure processing, and high-pressure homogenization on the physicochemical and functional properties of myofibrillar proteins[J]. Innov. Food Sci. Emerg. Technol..

[79] Shi X., Yang Y., Li Z. (2020). Moisture transfer and microstructure change of banana slices during contact ultrasound strengthened far-infrared radiation drying[J]. Innovative Food Science & Emerging Technologies.

[80] Bahwan M., Baba W.N., Adiamo O. (2023). Exploring the impact of various cooking techniques on the physicochemical and quality characteristics of camel meat product[J]. Animal Bioscience.

[81] Zhang R., Yuan J., Zhang W. (2024). Effects of ultrasound-assisted intermittent tumbling on the quality of cooked ham through modifying muscle structure and protein extraction[J]. J. Sci. Food Agric..

[82] Wang L., Li J., Teng S. (2022). Changes in collagen properties and cathepsin activity of beef *M. semitendinosus* by the application of ultrasound during post-mortem aging[J]. Meat Sci..

[83] Chang H.-J., Xu X.-L., Zhou G.-H. (2012). Effects of characteristics changes of collagen on meat physicochemical properties of beef *semitendinosus* muscle during ultrasonic processing[J]. Food Bioproc. Tech..

[84] Yin Y., Pereira J., Zhou L. (2020). Insight into the effects of sous vide on cathepsin B and L activities, protein degradation and the ultrastructure of beef[J]. Foods.

[85] Qi J., Li C., Chen Y. (2012). Changes in meat quality of ovine longissimus dorsi muscle in response to repeated freeze and thaw[J]. Meat Sci..

[86] Cheng H., Jung E.-Y., Song S. (2023). Effect of freezing raw meat on the physicochemical characteristics of beef jerky[J]. Meat Sci..

[87] Yang Y., Zhong J., Ma X. (2023). Effect of ultrasonic power on moisture migration and microstructure of contact ultrasound enhanced far-infrared radiation drying on taro slices[J]. Drying Technol..

[88] Li C.B., Zhou G.H., Xu X.L. (2010). Dynamical changes of beef intramuscular connective tissue and muscle fiber during heating and their effects on beef shear force[J]. Food Bioproc. Tech..

[89] Wattanachant S., Benjakul S., Ledward D.A. (2005). Effect of heat treatment on changes in texture, structure and properties of Thai indigenous chicken muscle[J]. Food Chem..

