# Supplementary material for: Effects of contact ultrasound coupled with infrared radiation on drying kinetics, water migration and physical properties of beef during hot air drying
Source: Ultrason Sonochem. 2024 Jul 3;108:106978. doi: 10.1016/j.ultsonch.2024.106978 (PMC11279329; doi:10.1016/j.ultsonch.2024.106978)
Supplement: Supplementary Data 1 [file mmc1.docx]

**Effects of contact ultrasound coupled with infrared radiation on drying kinetics, water migration and physical properties of beef during hot air drying.**

Jiahua Gao^1^, Siyu Cheng^2^, Xianming Zeng^1^, Xiaomei Sun^1^, Yun Bai^1^, Songmei Hu^1^, Jianping Yue^3^, Xiaobo Yu^1^, Minwei Zhang^4^, Xinglian Xu^1^, Minyi Han^*1, 2^

^1^ Key Laboratory of Meat Processing and Quality Control, Ministry of Education, Nanjing Agricultural University, Nanjing 210095, China

^2^ Wens Foodstuff Group Co., Ltd., Yunfu, 527400, China

^3^ Emin County Xinda Tongchuang Bioengineering Co., Ltd., Tacheng, 834600, China

^4^ Guangdong Testing Institute of Product Quality Supervision, Shunde, 528300, China

^*^Corresponding author, E-mail: [myhan@njau.edu.cn](mailto:myhan@njau.edu.cn)

**Supplementary methods**

Infrared absorption spectra

A defined quantity of fresh beef was homogenized by knife mill (HM100, Geruideman Instrument Equipment Co., Ltd., China), and a small aliquot was subsequently placed on a flat dish for freeze-drying. Upon completion of the drying process, 2 mg of the freeze-dried beef sample were accurately weighed and combined with 200 mg of dried KBr powder for grinding and tableting. A Fourier transform infrared spectrometer (Nicolet iS10, Thermo Fisher Scientific Corporation, USA) was utilized for data acquisition of the pressed tablets, and the spectral scanning range was set from 4000 to 400 cm^-1^ with a total of 64 scans executed at a resolution of 8 cm^-1^.

**Figures**





**Fig. S1.** Infrared absorption spectrum of beef.


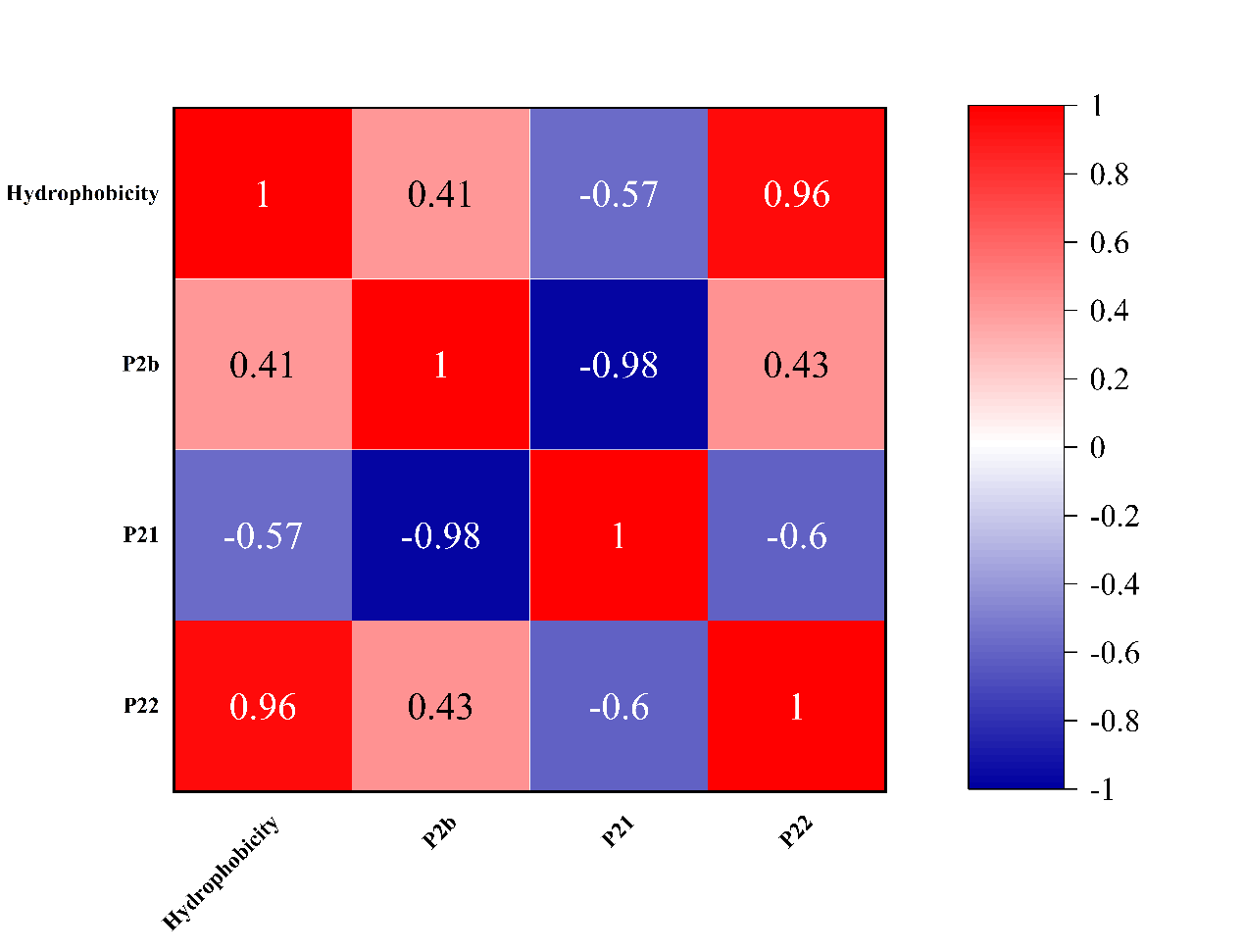


Fig. S2. Correlation analysis between surface hydrophobicity and water composition of air-dried beef.
